# Can Male Patient’s Age Affect the Cortical Bone Thickness of Jawbone for Dental Implant Placement? A Cohort Study

**DOI:** 10.3390/ijerph18084284

**Published:** 2021-04-18

**Authors:** Shiuan-Hui Wang, Yi-Chun Ko, Ming-Tzu Tsai, Lih-Jyh Fuh, Heng-Li Huang, Yen-Wen Shen, Jui-Ting Hsu

**Affiliations:** 1 School of Dentistry, China Medical University, Taichung 404, Taiwan; u104020415@cmu.edu.tw (S.-H.W.); Chip38916@hotmail.com (Y.-C.K.); ljfuh@mail.cmu.edu.tw (L.-J.F.); hlhuang@mail.cmu.edu.tw (H.-L.H.); 2 Department of Dentistry, China Medical University Hospital, Taichung 404, Taiwan; 3 Department of Biomedical Engineering, Hungkuang University, Taichung 433, Taiwan; anniemtt@sunrise.hk.edu.tw; 4 Department of Bioinformatics and Medical Engineering, Asia University, Taichung 413, Taiwan

**Keywords:** male, dental implant, dental cone-beam computed tomography, cortical bone thickness

## Abstract

Dental implants are among the most common treatments for missing teeth. The thickness of the crestal cortical bone at the potential dental implant site is a critical factor affecting the success rate of dental implant surgery. However, previous studies have predominantly focused on female patients, who are at a high risk of osteoporosis, for the discussion of bone quality and quantity at the dental implant site. This study aimed to investigate the effect of male patients’ age on the crestal cortical bone of the jaw at the dental implant site by using dental cone-beam computed tomography (CBCT). This study performed dental CBCT on 84 male patients of various ages to obtain tomograms of 288 dental implant sites at the jawbone (41 sites in the anterior maxilla, 95 in the posterior maxilla, 59 in the anterior mandible, and 93 in the posterior mandible) for measuring the cortical bone thickness. A one-way analysis of variance and Scheffe’s test were performed on the measurement results to compare the cortical bone thickness at implant sites in the four jaw areas. The correlation between male patient age and cortical bone thickness at the dental implant site was determined. The four jaw areas in order of the cortical bone thickness were as follows: posterior mandible (1.07 ± 0.44 mm), anterior mandible (0.99 ± 0.30 mm), anterior maxilla (0.82 ± 0.32 mm), and posterior maxilla (0.71 ± 0.27 mm). Apart from dental implant sites in the anterior and posterior mandibles, no significant correlation was observed between male patients’ age and the cortical bone thickness at the dental implant site.

## 1. Introduction

Dental implants are a popular treatment option for the replacement of missing teeth [1,2,3,4,5,6]. Titanium, due to its high biocompatibility, facilitates osseointegration between the dental implant and the jawbone, aiding in the fixation of the implant to the alveolar bone [1,7,8]. Jawbone quality and quantity can influence dental implant osseointegration ability and the failure rate of dental implant surgery [4,7,8,9,10]. The jawbone is constituted by porous cancellous bone on the inner side and by dense cortical bone on the outer side [5,6]. The literature has revealed a high correlation between the thickness of the cortical bone and the initial stability of dental implants; a high level of initial implant stability is conducive to osseointegration between the cancellous bone and implants [3,4,7,9].

Studies have uncovered a significant correlation between age and bone loss. According to the World Health Organization, “osteoporosis is present when the bone mass is more than 2.5 standard deviations (SD) below that of healthy premenopausal adult females, the T-score” [11]. Osteoporosis is categorized into two major types: primary osteoporosis and secondary osteoporosis [11,12,13,14,15]. Primary osteoporosis can be further categorized into two types: menopausal women experience rapid decline in the bone mass density due to estrogen reduction in Type I, and Type II is senile osteoporosis, which commonly occurs in people 60 years or older [11,14]. With age, vitamin D synthesis and calcium ion absorption into the gastrointestinal tract decrease, which can result in the development of secondary hyperparathyroidism, increase osteoclast activity, and accelerate bone loss [5,12,13,14].

Clinically, osteoporosis often leads to fractures in the hip bone and lumbar vertebrae. Iwasaki et al., 2012 [16] observed that osteoporosis increases the possibility of alveolar bone loss and tooth loss. Borrud et al., 2012 [13] found that women are four to six times more likely to develop osteoporosis than men. Studies have predominantly focused osteoporosis development in women. Men typically start to develop bone fractures in the hip, wrist, and lumbar vertebrae approximately 10 years later than women do. However, various researchers, including Alswat, 2017 [12], have shown that men are more prone to complications of osteoporosis. The mortality of men (37%) within 1 year of bone fractures was found to be 1.5 times higher than that of women [14]. The problem of missing teeth in older people has received increasing research attention, for which dental implant surgery is the common treatment. However, the survival rate of dental implants can be undermined if bone quality is low. Osteoporosis attributable to aging mainly involves reduced density of the cancellous bone. Nevertheless, few studies have explored the relationship between osteoporosis and the cortical bone [6], particularly cortical bone thickness in the jawbone.

The cortical bone of the jaw is among the most critical factors influencing the initial stability of dental implants [2,3,4,7,8,9,17]. The cortical bone of the jaw is thinner than that of all other body parts (e.g., cortical femur and tibia). Therefore, computed tomography (CT), which has low resolution, may fail to accurately determine the cortical bone thickness of the jaw due to the partial volume effect. By contrast, dental cone beam CT (dental CBCT) has a lower radiation dose and higher resolution, which contribute to its ability to more accurately measure the cortical bone thickness of the jaw and thus to its increasing adoption by dentists over the past few years.

Various studies investigating the relationship between age and bone quality in men have shown that aging can cause decreases in the bone density of the entire body. For men aged between 60 and 90 years, Chen et al., 2013 [18] revealed that, annually, the thickness of the cortical femur decreased by 3–5%, and cortical porosity increased by 31–33%. The survival rate of dental implants is affected by the strength of the jawbone at the sites of dental implants [19,20,21,22]. Additionally, the cortical bone of the jaw contributes greatly to the initial stability of implants. Several studies have discussed the effect of cortical bone thickness at the sites of dental implants on the efficacy of dental implant surgery in patients with missing teeth [5,6,23,24]. In our previous study, we revealed the effect of perimenopause on the cortical bone thickness at the sites of dental implants [24]. However, few studies have examined how age affects the quality of the jawbone or cortical bone thickness in men. Furthermore, due to the different movement patterns of and different forces applied to the mandible and maxilla, we inferred that the cortical bone thickness in the two parts of the jaw also decrease through different mechanisms. This study investigated the effect of age on the cortical bone thickness at the sites of dental implants in male patients by using dental CBCT.

## 2. Materials and Methods

### 2.1. Patient Selection and Cone-Beam Computed Tomography (CBCT) Scanning

In this study, 288 dental CBCT images of planned dental implant sites were collected from 84 male patients (average age: 50.5 ± 17.9 years) at the Dentistry Division of China Medical University Hospital between 2013 and 2016. The missing teeth were categorized according to their locations by four regions: 41 missing teeth were located in the anterior maxilla region, 95 in the posterior maxilla region, 59 in the anterior mandible region, and 93 in the posterior mandible region. The dental CBCT (AZ 3000, Asahi Roentgen, Japan) imaging parameters were set at 85 kV, 3 mA, and spatial resolution of 155 µm. This study was approved by the Institutional Review Board of China Medical University Hospital. All the patients were carefully evaluated by a dentist and judged to be suitable candidates for dental implants. The study is retrospective; most of the patients underwent dental CBCT scanning 4–6 months after tooth extraction. After scanning, they received dental implant placement.

### 2.2. Measurement Approach of Cortical Bone Thickness at Dental Implant Sites

**Figure 1:**
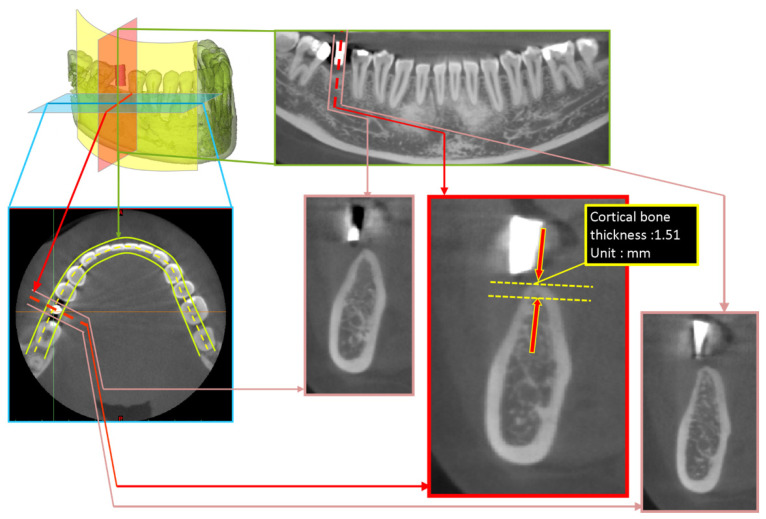
Measurement of cortical bone thickness at dental implant site.

The dental CBCT images were imported into the Digital Imaging and Communications in Medicine medical image software of Mimics 15.0 (Materialise, Leuven, Belgium). The images were resliced using the online reslice function in accordance with the shape of the dental arches to obtain the cross-sectional images of the dental arches. The gutta-percha indicators representing the best location for the implants can be used to obtain the position information of dental implant on the CBCT images. Crestal cortical bone thickness, which was made visible by radiopaque gutta-percha indicators, was then measured in the images (Figure 1).

### 2.3. Statistical Analysis

The cortical bone thickness measurement results are expressed as mean and standard deviation (SD; mean ± SD). (1) To examine whether the cortical bone thickness differed across jawbone regions (i.e., anterior maxilla, anterior mandible, posterior maxilla, and posterior mandible), one-way analysis of variance (ANOVA) was conducted, and Scheffe’s test was used for post hoc testing. (2) The Pearson correlation coefficient was employed to determine the correlation between age and the cortical bone thickness in male patients. All statistical analyses were performed using SPSS (IBM Corporation, Armonk, NY, USA).

## 3. Results

**Figure 2:**
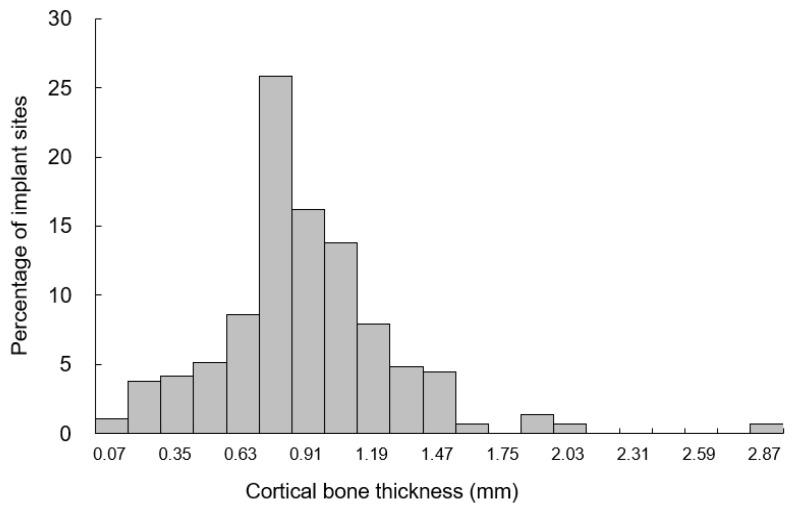
The distributions of crestal cortical bone thickness.

In this study, the thickness of the occlusal cortical bone at the planned dental implant sites was measured for 84 male patients; 288 measurements were obtained and are presented as mean ± SD. The cortical bone thickness was 0.89 ± 0.38 mm on average and ranged from 0.12 to 2.92 mm (Figure 2).

### 3.1. Cortical Bone Thickness at Dental Implant Sites

**Figure 3:**
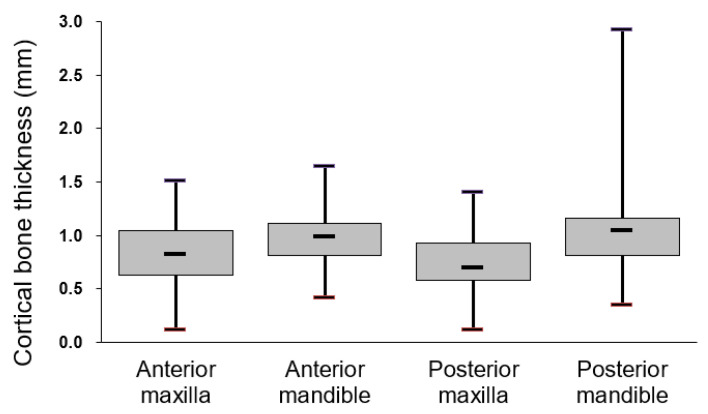
Cortical bone thickness at dental implant sites in different regions of the jawbone. Post-hoc Scheffe test results indicated significant differences (*p*  <  0.05) between the posterior mandible and the anterior maxilla, between the anterior mandible and the posterior maxilla, and between the posterior mandible and the posterior maxilla.

The cortical bone thickness of the four jawbone regions in descending order was as follows: posterior mandible: 1.07 ± 0.44 mm, anterior mandible: 0.99 ± 0.30 mm, anterior maxilla: 0.82 ± 0.32 mm, and posterior maxilla: 0.71 ± 0.27 mm. The cortical bone thickness of the four jawbone regions was subsequently compared in pairs, and only the following three pairs had significantly different thicknesses of cortical bone: posterior mandible (1.07 + 0.44 mm) > anterior maxilla (0.82 + 0.32 mm; *p* < 0.001) and anterior mandible (0.99 + 0.30 mm) > posterior maxilla (0.71 + 0.27 mm; *p* = 0.001) and posterior mandible (1.07 + 0.44 mm) > posterior maxilla (0.71 + 0.27 mm; *p* < 0.001; Figure 3).

### 3.2. Relationship between Age and Cortical Bone Thickness at Dental Implant Sites

**Figure 4:**
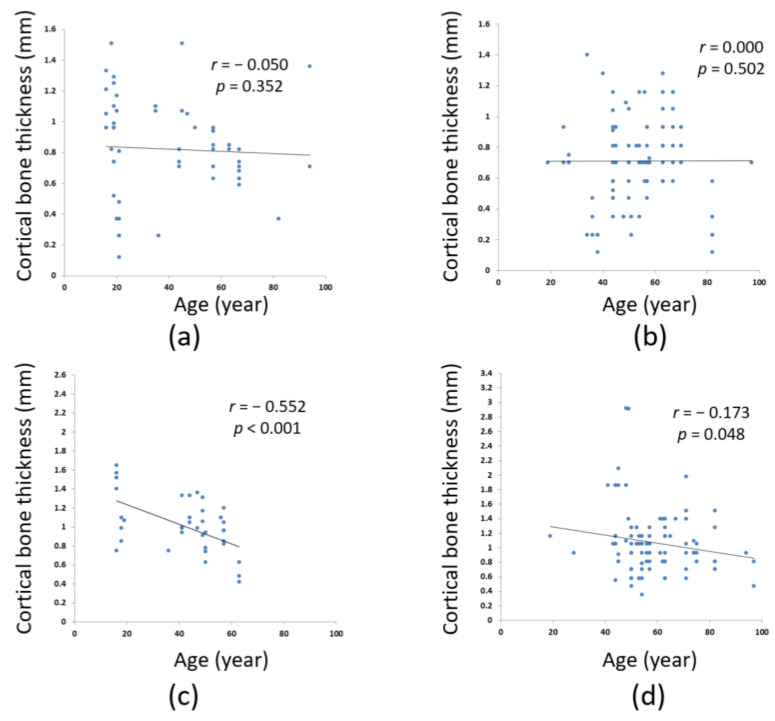
Relationships between cortical bone thickness at dental implant sites and age in the: (**a**) anterior maxilla; (**b**) posterior maxilla; (**c**) anterior mandible; (**d**) posterior mandible.

Figure 4 presents the correlation between age and the cortical bone thickness of different jawbone regions. No significant correlation was observed between age and cortical bone thickness in the anterior maxilla (Figure 4a) or posterior maxilla (Figure 4b; *p* > 0.05). Notably, age was moderately and negatively correlated (*r* = −0.552, *p* = 0.001) with cortical bone thickness in the anterior mandible region (Figure 4c) and weakly and negatively correlated (*r* = −0.173, *p* = 0.048) with that in the posterior mandible region.

## 4. Discussion

Population aging has led to an increasing prevalence of osteoporosis and missing teeth among older adults, both of which are critical issues affecting older adults’ quality of life. Dental implant surgery is used to restore occlusion function and replace missing teeth; in such surgery, bone quality and quantity at dental implant sites are crucial factors affecting the stability of the implants. Population aging has resulted in the increased occurrence of missing teeth. Recent studies have used dental CBCT to determine jawbone quality at the site of dental implants [10,25,26], but few studies have examined the relationship between age and jawbone quality in men; no study has investigated how age affects the cortical bone thickness at dental implant sites for men. Therefore, the present study explored the relationship between age and cortical bone thickness measured using dental CBCT before dental implant surgery in male patients, and the results revealed that the cortical bone thickness at the sites of mandibular dental implants, particularly in the anterior mandible region, decreased with age.

Various studies have uncovered a strong correlation of bone quality and quantity with the success rate of dental implantation [19,20,21,22]. Jemt and Lekholm, 1995 studied how the bone strength affects the failure rate of dental implantation and found that the rate was limited to 7.9% if bone quality at the planned implant sites was high; the rate increased to 28.8% if bone quality was low. Following the jawbone quality classification standards proposed by Lekholm and Zarb, 1985 [1], Jaffin and Berman, 1991 [19] investigated the relationship between the bone types of jaw regions with dental implants and the failure rate of the implantation. They investigated more than 1000 dental implants and found that bone type IV, which represented the lowest bone quality, was associated with a failure rate of 35%; for bone types I–III, the failure rate was limited to approximately 3%. High bone quality reduced the probability of the failure of dental implantation. Jawbone quality and quantity are typically determined through quantitative measurement of the cancellous bone density and cortical bone thickness [27], and the crestal cortical bone thickness of the jaw is closely related to the initial stability of dental implants. Miyamoto et al., 2005 [21] employed CT to measure the cortical bone thickness at the dental implant sites and proposed that the initial stability of dental implants is more affected by the cortical bone thickness than by the cancellous bone density.

Researchers have started using dental CBCT to determine jawbone quality and quantity, and it has increasingly replaced medical CT in dentistry due to its higher resolution, lower radiation dose, and lower cost. Although CBCT was previously considered unsuitable for the measurement of jawbone quality [28,29], advances in this technique in the last few years has led to its increasing use; an increasing number of studies have verified its suitability for measuring the cancellous bone density [23,24,26,30,31,32,33] and for evaluation and treatment simulation before dental implant surgery. According to Tsutsumi et al., 2011 [34], dental CBCT is appropriate for the measurement of the cortical bone thickness at the dental implant site if the thickness is three to four times greater than the voxel resolution of CBCT. Therefore, given that crestal cortical bone thickness measured in the present study was mostly three times greater than the resolution (155μm) of CBCT adopted in this study, dental CBCT was adequate for measuring the thickness of occlusal cortical bone at the sites of dental implants.

Studies that have measured the cortical bone thickness of the jaw by using dental CBCT have predominantly focused on the buccal and lingual cortical bone thicknesses at the sites of orthodontic mini implants [35,36,37]. Regarding research examining the crestal cortical bone thickness at the site of dental implants, Ko et al., 2017 [24] suggested the use of dental CBCT to measure the occlusal cortical bone thickness at different locations of the jaw; they observed the thickest cortical bone in the posterior mandible region, followed by the anterior mandible region, the anterior maxilla region, and the posterior maxilla region. Gupta et al., 2017 [23] found consistent results and verified the suitability of dental CBCT for measuring the cortical bone thickness of the jaw. The present study investigated male patients’ cortical bone thickness in dental implant sties and revealed the thickest cortical bone in the posterior mandible region, followed by the anterior mandible region, the anterior maxilla region, and the posterior maxilla region. According to the present study findings and those of the literature, the order of occlusal cortical bone thickness at the implant site among the aforementioned four jawbone regions is the same across sex and age. The reason for the thinnest cortical bone in the posterior maxilla region is that the maxillary sinus is located above the region, which may lead to pneumatization in the case of a missing tooth, in turn causing the overall bone quality of the posterior maxilla to decrease and the cortical bone to grow thinner.

In our previous study, we investigated the effect of age on the cortical bone thickness at the dental implant sites in female patients by dividing the patients into two groups based on the cut-off age of 50 years, which is the typical menopause age [5]. However, in the present study, male patients were not divided into age groups mainly because Type II senile osteoporosis is the most common osteoporosis type among older men, and this type of osteoporosis does not typically develop at a specific age. Additionally, the measurements of the cortical bone thickness obtained in the present study were all smaller than those obtained in previous studies using CT [21,38]; this is probably because CT has a lower resolution and thus tends to overestimate the cortical bone thickness if it is approximately or smaller than 1 m due to the partial volume effect. The CBCT method used in the present study had a 155 μm resolution, rendering it a more favorable alternative to CT in dentistry, in which high precision is required.

This study examined how age affects the crestal cortical bone thickness at the sites of dental implants in male patients and revealed no significant correlation between age and the bone thickness in the anterior or posterior maxilla region. However, age was correlated moderately and negatively with the thickness in the anterior mandible region (*r* = −0.552, *p* = 0.001) and weakly and negatively with that in the posterior mandible region (*r* = −0.173, *p* = 0.048). Thus, age was not associated with the crestal cortical bone thickness in the maxilla region, but that in the mandible region decreased with age. Chen et al., 2013 [18] explored cortical bone loss in different body parts of men and observed that as men aged, the cortical bone porosity of the radius increased, and the cortical bone strength decreased. In the same study, three stages of bone loss caused by aging were also established: cancellous bone loss caused by the microstructure of the trabecular bone growing thinner due to damage; cortical bone reduction caused by the increasing number of resorption cavities and increasing porosity attributable to aging; and continuous resorption at the cortical bone surface. Regarding studies related to bone loss in the maxilla and mandible, Abirami, 2016 [39] reported that the mandible shrank four times faster than the maxilla did. In a clinical observation report, Fanghänel et al., 2006 [40] proposed the different shrinkage patterns of the maxilla and mandible; specifically, the maxilla shrank mainly horizontally and the mandible vertically. Woelfel et al., 1976 [41] asked patients to perform occlusion movement under a force of 50 pounds was applied and observed how the force was distributed to each region of the jaw; they found that the amount of force distributed to the mandible (21 pounds/inch) was approximately twice as high as that to the maxilla (12 pounds/inch). Due to such different amounts of force applied to the maxilla and mandible during occlusion movement, they concluded that the bone loss rate may differ in different regions of the jaw. Accordingly, the present study inferred that the different force distribution to and the different structures of the maxilla and mandible were the reason why they shrank along different directions, and the greater force applied to the mandible was probably why the mandible was more susceptible to senile osteoporosis with regards to cortical bone thinning. The present study results revealed that the cortical bone thickness in the maxilla did not decrease significantly with age in male patients, which was possibly because the maxilla, to which force is constantly applied, is constituted mostly by cancellous bone. Furthermore, according to the findings of D’Souza, 2012 [42], the trabecular bone of the maxilla is parallel to the direction of compression and deformation; thus, sudden stress increases can be alleviated in the bone and the bone is thus resilient to deformation. Cancellous bone is porous and contains a large amount of vascular tissue. Compared with the mandible, the maxilla is composed of more cancellous bone and thus has higher blood supply. Therefore, the cortical bone thickness reduction in the maxilla attributable to senile osteoporosis is less obvious than that in the mandible.

Numerous clinical factors can affect cortical bone thickness at the dental implant sites of the jawbone. Most of the samples in this study were collected using CBCT within 4–6 months after dental extraction. Therefore, the study design excluded the effect of a missing tooth on cortical bone thickness. In addition, the study did not discuss a number of conditions. Regarding drug treatment, patients taking bisphosphonate drugs were not recommended to undergo invasive treatments such as tooth extractions or dental implants. Therefore, among the implant patients, the number of individuals in this category was small. Regarding systemic disease, osteoporosis generally does not have a clear time point for occurrence in male patients. Therefore, fewer male patients proactively undergo bone testing, and clinical determination of osteoporosis incidence in male patients is difficult. With regard to tumor diseases, the disease more relevant to this study should be bone tumors of the jaw. Such patients are treated with metal bone screws or bone plates after tumor resection for fixation. Hence, these patients were excluded during image screening. Regarding risk factor, habits such as smoking and drinking may affect the bone quality and quantity of the jawbone. Nonetheless, risk factors were not recorded because the study was retrospective. If subgroup investigations including lifestyle habits are conducted in the future, the results of this study can be used as a reference.

This study has the following limitations. First, the study participants are all Asian and ethnically Mongolian. Therefore, further research is required to examine whether the distribution of cortical bone thickness is consistent across races. Additionally, this study compared age with the cortical bone thickness in men only through correlation analysis, without grouping male patients by their type of osteoporosis. Finally, the study is a retrospective analysis of bone quality and quantity of the dental implant site. Therefore, information on whether the patients had several treatments, systemic conditions, or local risk factors was unavailable. In the future, the research team will attempt to explore the effect of Type II osteoporosis on the thickness of the occlusal cortical bone in the jaw as well as on the initial and long-term stability of dental implants. Future studies can include subgrouping by, for example, smoking, drinking, and long-term medication in addition to age to study crestal cortical bone thickness in the dental implant site, providing more valuable clinical reference data.

## 5. Conclusions

This study proposed the following conclusions regarding the relationship between age and the crestal cortical bone thickness at dental implant sites in male patients:

(1) Male patients were grouped by the jawbone regions where their dental implants were placed. The cortical bone thickness of the jawbone regions in descending order was as follows: posterior mandible: 1.07 ± 0.44 mm, anterior mandible: 0.99 ± 0.30 mm, anterior maxilla: 0.82 ± 0.32 mm, and posterior maxilla: 0.71 ± 0.27 mm.
(2) The cortical bone thickness at dental implant sites in the maxilla did not differ across age in male patients.
(3) Among male patients, age was correlated moderately and negatively with the cortical bone thickness in the anterior mandible region (*r* = −0.552, *p* = 0.001) and weakly and negatively with the posterior mandible region (*r* = −0.173, *p* = 0.048).

## Data Availability

The data used to support the findings of this study are available from the corresponding author upon request.
